# Jian-Kang Liu: A pioneer of sex determination studies in vertebrates

**DOI:** 10.1007/s13238-015-0232-7

**Published:** 2015-12-19

**Authors:** Li Zhou, Jian-Fang Gui

**Affiliations:** State Key Laboratory of Freshwater Ecology and Biotechnology, Institute of Hydrobiology, Chinese Academy of Sciences, Wuhan, 430072 China

**Figure 1:**
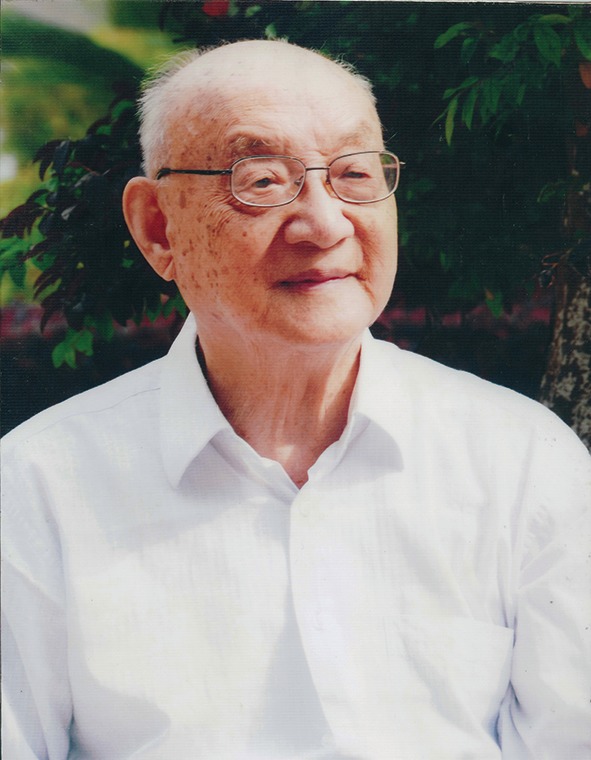
Dr. Jian-Kang Liu in 2014

Sixty-eight years ago, Dr. W.S. Bullough, a Fellow of the Royal Society, published an article “Hermaphroditism in the lower vertebrates” in *Nature* (Bullough, 1947) and thereby commented a significant finding in lower vertebrate sex mechanism fulfilled by a young Chinese scholar Jian-Kang Liu (刘建康, C.K. Liu) (Fig. 1). At the beginning of the article, Dr. Bullough respectfully acknowledged that: “By the publication in 1944 of a description of the gonads of *Monopterus javanensis* Lac. (Symbranchii; Teleostei) (Liu, 1944a), Liu has furnished new and interesting evidence concerning the mechanism of sex determination in the lower vertebrates, and has opened a fresh field for research into this subject” (Bullough, 1947).

**Figure 2:**
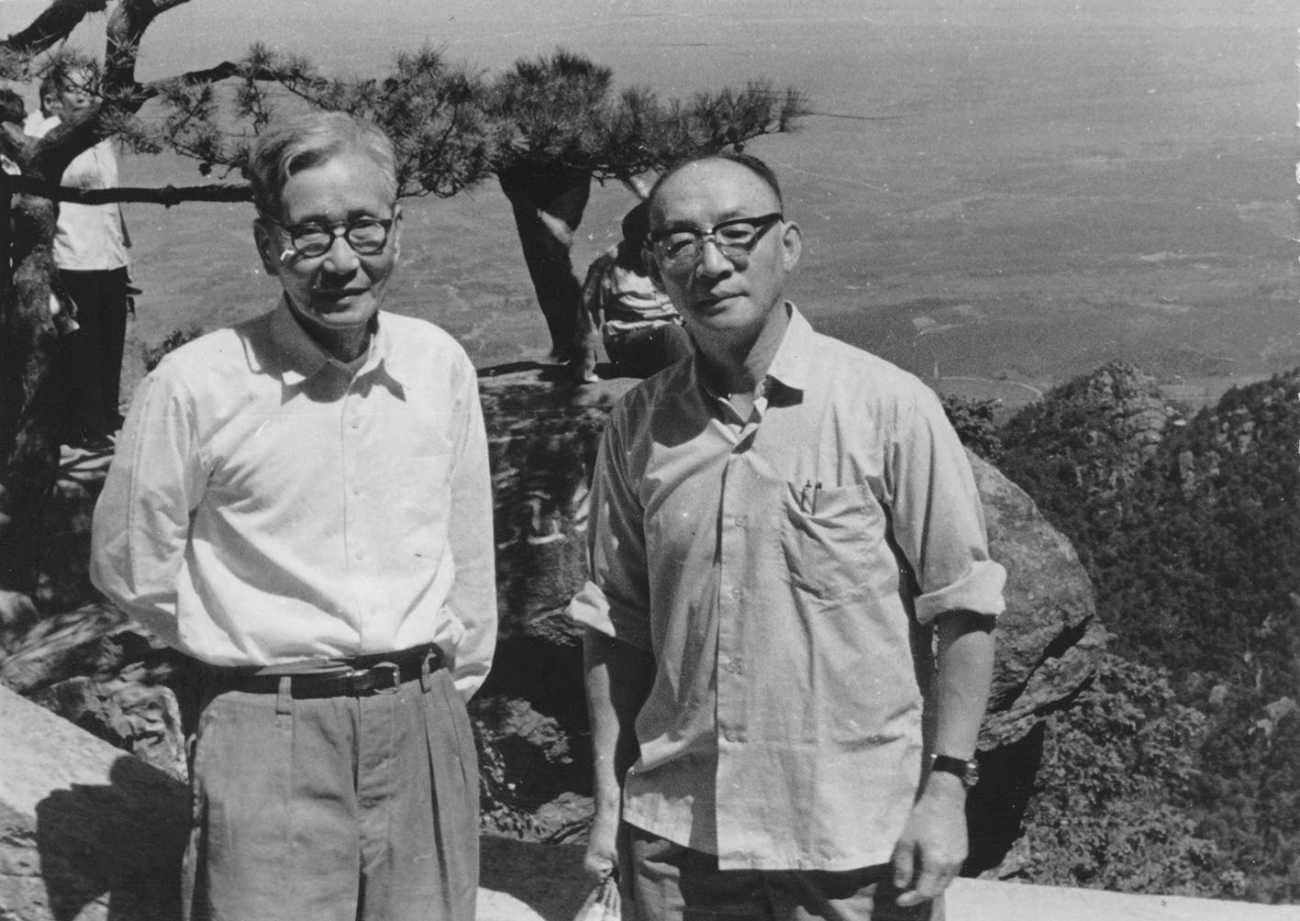
Dr. Jian-Kang Liu (front right) with his advisor Dr. Xian-Wen Wu (front left) in 1979

**Figure 3:**
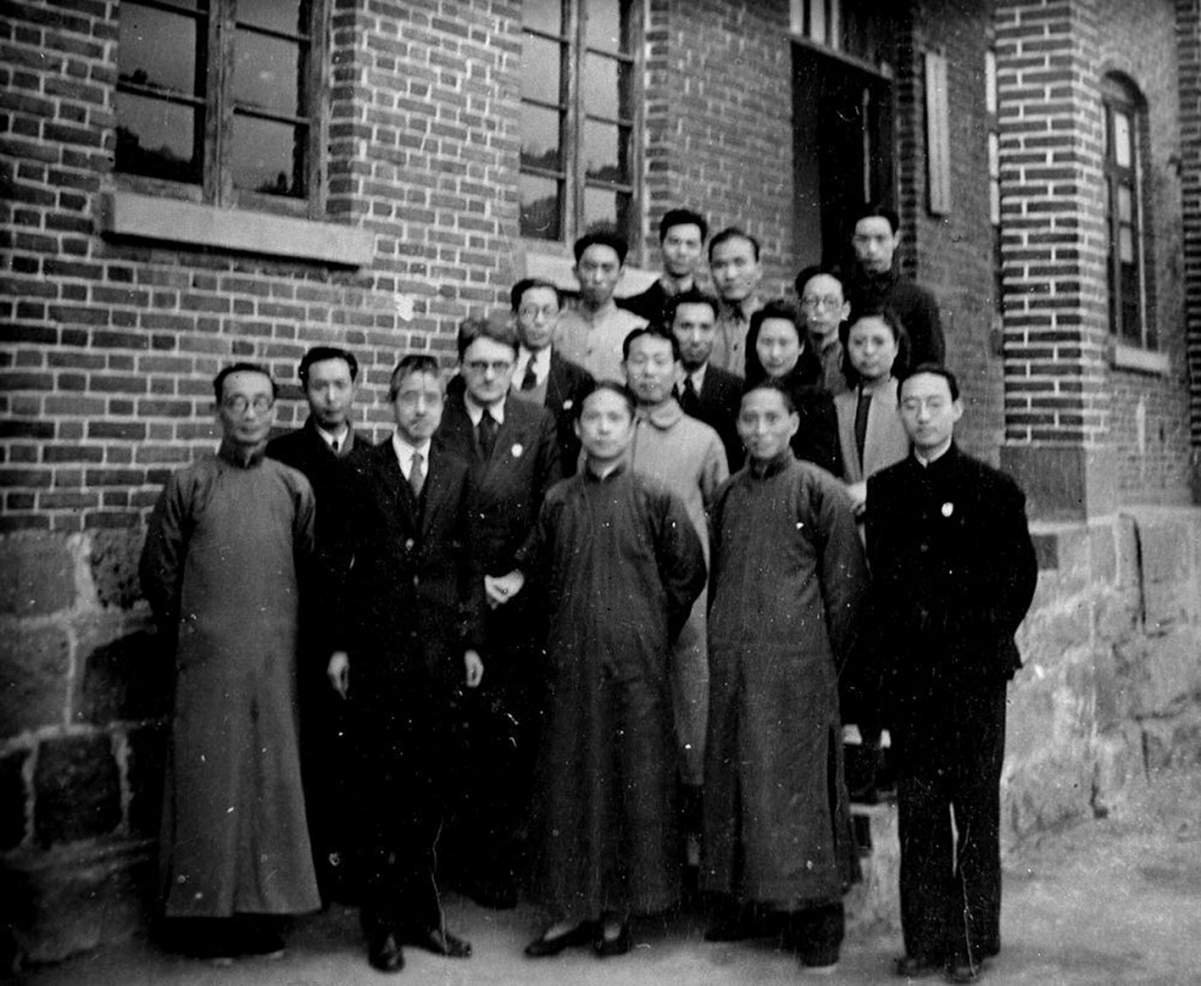
Dr. Jian-Kang Liu (The first at the front from right), Prof. Joseph Needham (The 2nd at second raw from left), and the principal investigators in Institute of Zoology and Botany of Academia Sinica in 1943

Jian-Kang Liu was born in Wujiang County, Jiangsu Province in 1917. In 1938, he graduated from the Biology Department of Soochow University, and accepted an offer from Institute of Zoology and Botany of Academia Sinica and began his innovative studies on fish biology directed by famous ichthyologist Dr. Xian-Wen Wu (伍献文) (Fig. 2). At that duration of the Anti-Japanese War, the Institute was forced to move from Nanjing to Guangxi, Yangshuo, Changsha, Nanyue, and finally to arrive Chongqing in 1939. Under the difficult conditions, as a young scholar, Jian-Kang Liu studied very hard and became the first to notice an unusual phenomenon about the sexuality of rice field eel (*Monopterus javanensis*) and obtained his early most remarkable achievement when he was only 27 years old. He carefully examined the gonads of 659 specimens ranging from 5.3 to 57.6 cm in total length and revealed hermaphroditism and sex reversal in the lower vertebrate (Liu, 1944a). After the paper was published, Dr. Bullough introduced the interesting findings in his lecture at Utrecht University in Netherlands and commented its scientific significance in *Nature* (Bullough, 1947). At the same time, Jian-Kang Liu also paid his attention to the development of fish secretory cells. He found that Sodium Sulphate can induce the development of “chloride-secreting cells” in *Macropodus* besides salinity (Liu, 1942). In 1943, Prof. Joseph Needham at Cambridge University accepted an assignment of the British Council for Cultural exchange with other Countries and cooperated with Institute of Zoology and Botany of Academia Sinica (Fig. 3). Under Prof. Joseph Needham’s recommendation, this work also published in *Nature* as a letter to editor (Liu, 1944b). From 1939 to 1945, young Jian-Kang Liu published a total of 19 research articles (Gui, 2007). From 1946 to 1947, he continued his research with Prof. N.J. Berrill at McGill University of Canada, and obtained his Ph. D. degree. After working for two years in USA, he returned to Shanghai, and was offered a faculty position at the current Institute of Hydrobiology, Chinese Academy of Sciences. Since then, Dr. Liu has developed into a well-known fish biologist and freshwater ecologist, and become a former and honorary director of the Institute. In 1981, he was elected as an Academician of the Chinese Academy of Sciences.

As Dr. Bullough said, the discovery of sex reversal in rice field eel indeed opened a fresh field for sex determination study after several decades in vertebrates, especially in fishes. Up to the present day, a large number of significant breakthroughs have been achieved from the genetic basis to the biotechnological manipulation of fish sexual dimorphism and sex determination (Gui and Zhu, 2012; Gui, 2015a; Mei and Gui, 2015). Because some aquaculture fishes exhibit significant sexual dimorphism in growth rate and body size, there are remarkable economic benefits by producing all-females or all-males for aquaculture, and several generations of fish genetic breeding scientists in the Institute have developed a series of sex control breeding biotechnologies to obtain mono-sex populations. Through heterologous sperm-induced gynogenesis and various reproduction mode utilization, three new unisexual all-female varieties, such as allogynogenetic gibel carp (Jiang et al., 1983), high dorsal allogynogenetic gibel carp (Zhu and Jiang, 1993), and allogynogenetic gibel carp “CAS III” (Wang et al., 2011), were bred in gibel carp and applied to aquaculture practice throughout China (Gui and Zhou, 2010; Gui and Zhu, 2012). By artificial gynogenesis and chromosome set manipulation, all-female hybrid common carp was also produced (Wu et al., 1986). Moreover, some sex-specific or sex chromosome-specific genetic markers were identified from yellow catfish and other bagrid catfish (*Pseudobagrus ussuriensis*) (Wang et al., 2009; Dan et al., 2013; Pan et al., 2015), and all-males of yellow catfish had been massively produced and used for commercial aquaculture (Liu et al., 2013; Gui, 2015b).

As a pioneer of sex determination studies in vertebrates and a founder of freshwater ecology in China, Dr. Liu has made significant contributions to fish biology, lake ecology, and sustainable aquaculture industry. As his fellow scholars, we would like to express our deepest gratitude for his leading role as a front-runner of sex determination studies in fish and express our devout wishes for his health and for his long life.
